# Citalopram-induced pathways regulation and tentative treatment-outcome-predicting biomarkers in lymphoblastoid cell lines from depression patients

**DOI:** 10.1038/s41398-020-00900-8

**Published:** 2020-07-01

**Authors:** Abdul Karim Barakat, Catharina Scholl, Michael Steffens, Kerstin Brandenburg, Marcus Ising, Susanne Lucae, Florian Holsboer, Gonzalo Laje, Ganna V. Kalayda, Ulrich Jaehde, Julia Carolin Stingl

**Affiliations:** 1 Federal Institute for Drugs and Medical Devices (BfArM), Bonn, Germany; 2 Department of Clinical Pharmacy, University of Bonn, Bonn, Germany; 3 Max Planck Institute of Psychiatry, Munich, Germany; 4 Washington Behavioral Medicine Associates LLC, Chevy Chase, MD USA; 5 Institute of Clinical Pharmacology, Faculty of Medicine, RWTH Aachen University, Aachen, Germany

**Keywords:** Predictive markers, Medical genetics, Depression

## Abstract

Antidepressant therapy is still associated with delays in symptomatic improvement and low response rates. Incomplete understanding of molecular mechanisms underlying antidepressant effects hampered the identification of objective biomarkers for antidepressant response. In this work, we studied transcriptome-wide expression followed by pathway analysis in lymphoblastoid cell lines (LCLs) derived from 17 patients documented for response to SSRI antidepressants from the Munich Antidepressant Response Signatures (MARS) study upon short-term incubation (24 and 48 h) with citalopram. Candidate transcripts were further validated with qPCR in MARS LCLs from responders (*n* = 33) vs. non-responders (*n* = 36) and afterward in an independent cohort of treatment-resistant patients (*n* = 20) vs. first-line responders (*n* = 24) from the STAR*D study. In MARS cohort we observed significant associations of *GAD1* (glutamate decarboxylase 1; *p* = 0.045), *TBC1D9* (TBC1 Domain Family Member 9; *p* = 0.014–0.021) and *NFIB* (nuclear factor I B; *p* = 0.015–0.025) expression with response status, remission status and improvement in depression scale, respectively. Pathway analysis of citalopram-altered gene expression indicated response-status-dependent transcriptional reactions. Whereas in clinical responders neural function pathways were primarily up- or downregulated after incubation with citalopram, deregulated pathways in non-responders LCLs mainly involved cell adhesion and immune response. Results from the STAR*D study showed a marginal association of treatment-resistant depression with *NFIB* (*p* = 0.068) but not with *GAD1* (*p* = 0.23) and *TBC1D9* (*p* = 0.27). Our results propose the existence of distinct pathway regulation mechanisms in responders vs. non-responders and suggest *GAD1, TBC1D9*, and *NFIB* as tentative predictors for clinical response, full remission, and improvement in depression scale, respectively, with only a weak overlap in predictors of different therapy outcome phenotypes.

## Introduction

The molecular pathophysiology of depression is to date not fully deciphered nor are the molecular mechanisms of antidepressant effects. This intricacy of depression has resulted in suboptimal treatment outcomes, leaving one to two-thirds of patients without adequate response to the first prescribed treatment^1^. Concrete means to predict clinical response are lacking. Genome-wide association studies (GWAS) conducted so far have not established solid predictors for antidepressant response^2–4^. Therefore, research on factors other than stable germline genetic variants explaining clinical response are currently in focus. Peripheral expression biomarkers have been subject of extensive research due to sampling feasibility in comparison to brain tissues. Peripheral gene expression was found to have significant similarities with multiple CNS tissues. In a study of transcriptional profiling of 79 human tissues for 33,698 genes the group median correlation between whole blood and 16 CNS tissues was intermediate (*ρ* = 0.52) lying higher than the correlation with muscle and peripheral nervous tissues (*ρ* = 0.48, 0.36, respectively) but below that with the immune system (*ρ* = 0.64)^5^. Consequently, expression profiling in blood cells has emerged as a promising tool for the identification of markers for psychiatric diseases^6,7^. EBV-transformed lymphoblastoid cell lines (LCLs) were shown to reliably maintain inter-individual variation in gene-expression levels^8^, resulting in their successful use in antidepressant response-predicting biomarkers research. In earlier studies, we investigated baseline gene expression of target genes in LCLs from patients with depressive episodes and identified *ITGB3* and *CHL1* to be candidate predictive biomarkers for clinical remission i.e. full clinical recovery^9^. Recently, we investigated functional neuroplasticity biomarkers by studying ex vivo proliferation rates and gene expression profiles of LCLs from patients with depressive episodes after long-term (21-day) in-vitro incubation with fluoxetine. Cell proliferation was found to correlate with treatment resistance i.e. when comparing responders to treatment-resistant patients, but not with response, i.e. when comparing responding to non-responding patients. Additionally, we identified transcriptional biomarkers for treatment resistance and response. Expression levels of *WNT2B, ABCB1*, and *FZD7* were found predictive for treatment resistance while expression of *WNT2B, SULT4A* correlated with response suggesting *WNT2B* as in common predictor^10,11^.

The aim of the current study was to identify candidate transcriptional response biomarkers in LCLs from stratified patients specifically diagnosed with major depressive disorder (MDD) and treated with serotonin-transporter-inhibiting antidepressants. We compared transcriptional profiles in responding and non-responding patients LCLs upon short-term incubation with the serotonin-selective reuptake inhibitor citalopram. Candidate genes were determined using whole-transcriptome analysis of LCLs from an exploratory cohort of SSRI-treated patients from Munich Antidepressant Response Signature (MARS) study. Differences in deregulated pathways and functional characteristics of asymmetrically expressed candidate genes were identified. Validation of candidate genes was done in a larger MARS cohort. Moreover, in an independent analysis, we investigated the candidate genes for associations with therapy resistance status in LCLs from response edge groups i.e. first-line responders and treatment-resistant patients recruited in the Sequenced Treatment Alternatives to Relieve Depression (STAR*D) study. Our results propose the existence of distinct pathway regulation mechanisms in responders vs. non-responders and suggest *GAD1, TBC1D9*, and *NFIB* as tentative predictors for clinical response, full remission, and improvement in depression scale, respectively, with only a weak overlap in predictors of different therapy outcome phenotypes.

## Materials and methods

### Cell lines and study population

#### Munich Antidepressant Response Signature (MARS) cohort

MARS study was an observational, open-label clinical study with the aim to analyze pharmacogenetics of therapy response in hospitalized patients with depressive episodes^12^. Patients were treated according to the psychiatrist choice for 8 weeks and were interviewed on a weekly basis to document therapy and clinical progress. Depression severity was measured using the 21-item Hamilton depression score (HAMD). Clinical response and clinical remission were defined as ≥50% reduction in baseline HAMD score, and as a score <8, respectively. EDTA blood samples from 150 MARS patients were received from Max-Plank-Institute for Psychiatry and underwent transformation with Epstein-Barr virus in the German Federal Institute for Drugs and Medical Devices (BfArM) as previously described^11^. Transformation to LCLs was successful in 144 samples with success rate of 96%.

**Fig. 1:**
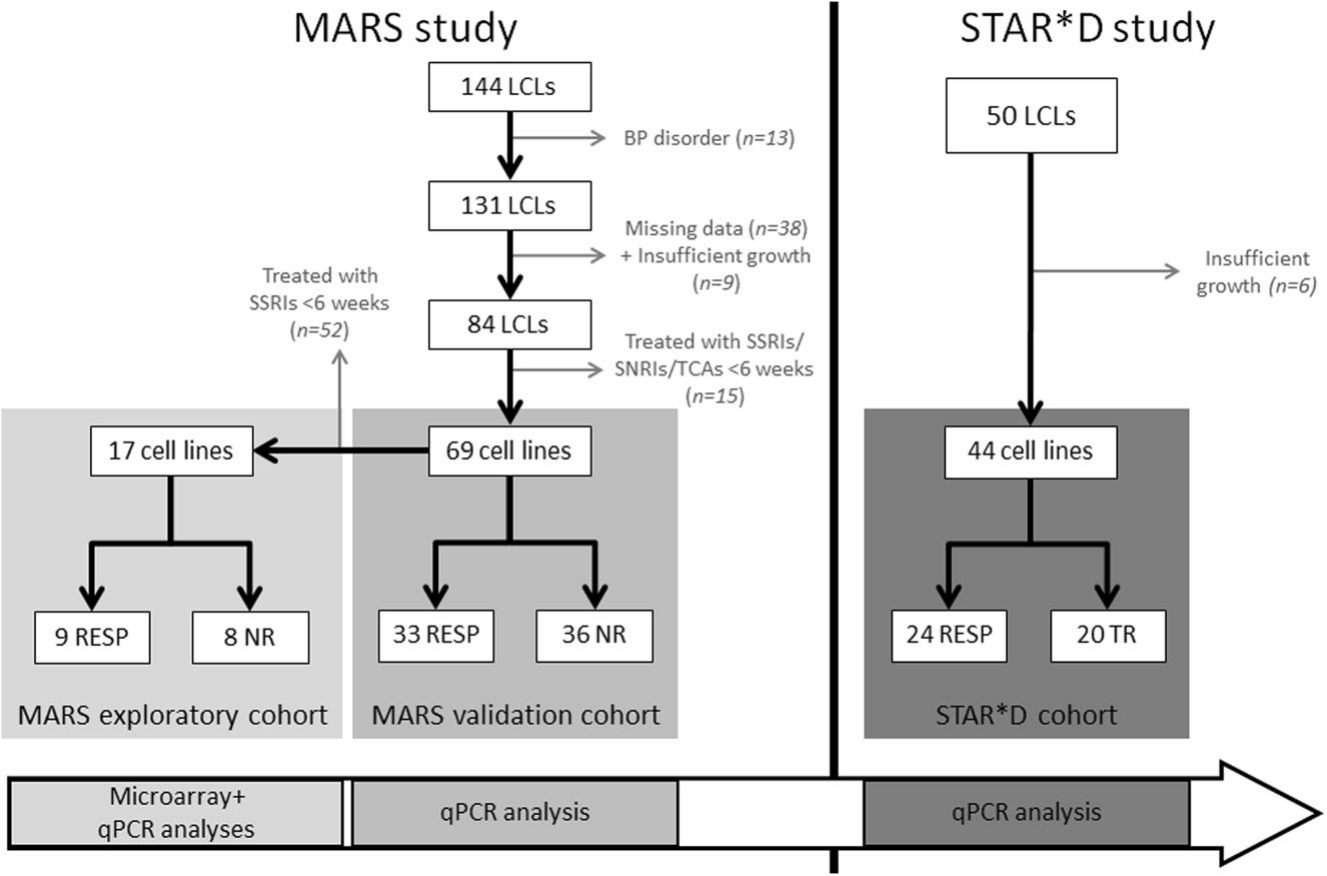
Stratification chart of LCLs derived from depression patients of MARS and STAR*D studies. MARS LCLs were stratified according to the diagnosis and clinical treatment profiles of the donor patients to obtain a homogenously SSRI-treated “exploratory cohort” and a “validation cohort” of SSRI-, SNRI- and TCA-treated patients. LCLs purchased from the STAR*D study were derived from 24 first-line-treatment responders and 20 treatment-resistant patients. Study setup is depicted as experimental steps done on each cohort. LCLs lymphoblastoid cell lines, RESP responding patient, NR non-responding patient, TR treatment-resistant patient, qPCR quantitative PCR.

To obtain a homogenous cohort for investigating biomarkers for response to serotonin-transporter-inhibiting antidepressants, donor patients were stratified on their clinical diagnosis and therapy profiles. Bipolar disorder patients (*n* = 13), patients with missing data at more than two visits (*n* = 38) and cell lines with insufficient growth (*n* = 9) were excluded. Patients were included if had been treated with at least one serotonin-transporter-inhibiting antidepressant form the classes: serotonin selective reuptake inhibitors (SSRIs), serotonin norepinephrine reuptake inhibitors (SNRIs) or tricyclic antidepressants (TCAs) for ≥6 weeks. A cohort of 69 LCLs: 36 non-responding (NR) and 33 responding patients (RESP), of which 16 were full remitters resulted and was designated the “validation cohort”. Out of this cohort, an exploratory cohort of SSRI-treated patients-derived LCLs (*n* = 17, 9 RESP, 8 NR) was used for RNA-microarray analysis of candidate genes, which were later validated in the validation cohort (Fig. 1, Table 1a; Supplementary Tables 1, 2).

**Table 1:** Clinical characteristics of MARS exploratory and validation cohorts (Table 1a) and STAR*D cohort (Table 1b).

Table 1a								
	MARS exploratory cohort				MARS validation cohort			
	Total (*n* = 17)	Responders (*n* = 9)	Non-responders (*n* = 8)	*p*-value	Total (*n* = 69)	Responders (*n* = 33)	Non-responders (*n* = 36)	*p*-value
Gender (females)	11	3	8	0.01	33	13	20	N.S
Age	42.53 ± 12.43	38.11 ± 12.48	47.50 ± 11.02	N.S	47.46 ± 14.18	46.55 ± 15.16	47.39 ± 13.44	N.S
HAMD baseline	24.12 ± 6.24	25.78 ± 6.82	22.25 ± 5.34	N.S	25.62 ± 6.37	26.21 ± 5.81	25.08 ± 6.88	N.S
HAMD 8 weeks	12.65 ± 7.15	8.11 ± 4.01	17.75 ± 6.50	0.004	13.06 ± 6.95	7.70 ± 4.47	17.97 ± 4.87	9.35E−14
Diagnosis								
Depressive episode (ICD10 code F32)	8	6	2	N.S	20	11	9	N.S
Recurrent depressive disorder (ICD10 code F33)	9	3	6		49	22	27	
Treatment duration (weeks)								
TCAs	0.41 ± 1.70	0.00 ± 0.00	0.88 ± 2.47	N.S	2.52 ± 3.46	2.06 ± 3.31	2.94 ± 3.59	N.S
SSRIs	7.59 ± 0.71	7.78 ± 0.67	7.38 ± 0.74	N.S	2.91 ± 3.48	3.79 ± 3.79	2.11 ± 3.01	0.05
SNRIs	0.59 ± 1.50	0.11 ± 0.33	1.13 ± 2.10	N.S	3.65 ± 3.65	3.18 ± 3.63	4.08 ± 3.66	N.S
NASSAs	1.06 ± 2.11	0.44 ± 0.88	1.75 ± 2.87	N.S	0.90 ± 2.14	1.39 ± 2.61	0.44 ± 1.50	N.S
NARIs	0.12 ± 0.49	0.22 ± 0.67	0.00 ± 0.00	N.S	0.22 ± 1.07	0.12 ± 0.48	0.31 ± 1.41	N.S
SSRE	0.00 ± 0.00	0.00 ± 0.00	0.00 ± 0.00	N.S	0.00 ± 0.00	0.00 ± 0.00	0.00 ± 0.00	N.S
Other ADs	0.24 ± 0.97	0.00 ± 0.00	0.50 ± 1.41	N.S	1.13 ± 2.27	1.00 ± 2.41	1.25 ± 2.17	N.S
Antipsychotics	3.47 ± 3.36	3.44 ± 3.09	3.50 ± 3.85	N.S	4.23 ± 3.54	3.73 ± 3.58	4.69 ± 3.50	N.S
Mood stabilizers	2.88 ± 3.04	2.56 ± 2.74	3.25 ± 3.49	N.S	2.78 ± 3.09	2.58 ± 2.86	2.97 ± 3.32	N.S
Benzodiazepines	3.59 ± 3.02	2.89 ± 3.44	4.38 ± 2.45	N.S	3.35 ± 3.07	3.70 ± 3.19	3.03 ± 2.96	N.S
Sedatives	1.29 ± 1.76	1.56 ± 2.24	1.00 ± 1.07	N.S	1.17 ± 1.74	1.06 ± 1.80	1.28 ± 1.70	N.S
Total drug-treatment weeks	21.24 ± 7.95	19.00 ± 7.47	23.75 ± 8.19	N.S	22.87 ± 7.70	22.61 ± 7.77	23.11 ± 7.75	N.S

Table 1b								
					Total (*n* = 44)	Responders (*n* = 24)	Treatment-resistants (*n* = 20)	*p*-value
Anxious depression					27	14	13	N.S
Gender								
Females					23	14	9	N.S
Males					21	10	11	
Age					48.09 ± 12.13	47.54 ± 13.65	48.75 ± 10.32	N.S
QIDS 0					17.66 ± 3.06	16.92 ± 3.08	18.55 ± 2.86	N.S
QIDS end					7.95 ± 6.73	2.46 ± 1.89	14.55 ± 3.78	3.70E−13

Differences were tested with Fisher test for gender and diagnosis and with *t*-test for age, depression scores and treatment duration (*p* ≤ 0.05). Data are shown as mean ± SD. N.S not significant.

#### Response edge groups of Sequenced Treatment Alternatives to Relieve Depression (STAR*D) study

STAR*D was an interventional, multi-center, randomized controlled clinical study on response genetics in major depression disorder (MDD) outpatients^13^. Patients were treated according to a sequential 4-level-treatment protocol. At level 1 all patients were treated with individually adjusted doses of citalopram. Patients without response or with severe treatment-intolerance in each level entered the next level where the therapy was switched or augmented with additional psychotropic drugs or with psychotherapy. Disease severity was assessed using QIDS score^14^ along 14 weeks in 2–3-week intervals. Clinical response was defined as ≥50% reduction in baseline score. In the current work, LCLs (*n* = 50) collected by G. Laje were purchased from NIMH Center for Collaborative Genetic Studies, Rodgers repository (Bethesda, MD, USA). Included LCLs were derived from response edge groups of the STAR*D patients with 24 first-level responders and 20 treatment-resistant patients (level-4-non-responders; Fig. 1, Table 1b, Supplementary Table 3). Six LCLs had insufficient growth to be included in the analysis. Analyses in the STAR*D cohort aimed to test the expression of the candidate genes for association with treatment resistance as a distinct clinical outcome phenotype. LCLs acquisition, culture, and qPCR validation are provided in Supplementary Methods 1.

### Genome-wide expression analysis

Whole-genome expression analyses were done using Agilent Single Color platform of 8 × 60 K microarrays. Data were analyzed using GeneSpring software (v.14.1.9, Agilent). Signal quality was maintained by filtering probes with compromised flags out. Differential gene expression was calculated in fold-change (FC) in sets of pairs: non-responders vs. responders (NR/RESP) or citalopram vs. control (CTP/ctrl., in both RESP and NR groups). Pathway analysis was done in pathway database imported from GenMAPP Pathway Markup Language^15^.

Gene-expression datasets from the exploratory MARS cohort (*n* = 17) were analyzed using a hypothesis-free algorithm designed to detect cardinal differences between the two response subgroups. Here, genetic features coding for autosomal genes and being over 2-fold differentially expressed in NR vs. RESP (FC ≥ 2) with Benjamini–Hochberg-corrected *p* value (*p*-corr. ≤ 0.05) under CTP incubation were determined at each time point, 24 and 48 h. Features with consistently differential expression (FC ≥ 2 NR/RESP) at both time points were further considered. Cardinality was ensured by computing a mean FC value (NR/RESP) over both incubations at both time points for each gene. Top ten features ranked for mean FC were considered candidate for qPCR validation.

A further, independent pathway-guided algorithm was designed to detect reactional transcriptomic differences between the response groups. Here, changes in gene expression before and after CTP incubation were analyzed by identifying genetic features that reacted to CTP incubation (hence, reactional) with a FC ≥ 2 (CTP/ctrl.) in RESP and NR groups at each time point, 24 and 48 h. These features underwent pathway-enrichment analysis (*p-*corr. ≤ 0.05) to determine altered functions in each response group. Features enriched in responders’ pathways were identified and filtered on FC cutoff NR/RESP ≥ 2. It is noteworthy that changes in gene expression before and after CTP incubation were not considered in the hypothesis-free algorithm.

### Statistical analysis

Descriptive analysis of the study cohorts was done using Fisher exact test and Student’s *t*-test. Gene expression data obtained from microarray experiments were analyzed using the statistical tools in GeneSpring software. Here, differences in gene expression were tested for significance using two-way ANOVA test adjusted for multiple testing using Benjamini–Hochberg FDR (*p*-corr. ≤ 0.05). *P*-values for pathways were computed using a hypergeometric computation intrinsically corrected for number of genetic features in the microarray, in genes of interest and of matched features in a pathway. qPCR results were tested for association with response and remission status in MARS population and with treatment-resistance in the independent STAR*D cohort using unpaired Welch’s *t*-tests (unadjusted *p* ≤ 0.05). Furthermore, ∆Cp values were tested for correlations with clinical improvement, calculated as HAMD_8w_/HAMD_baseline_ and QIDS_end_/QIDS_baseline_, using Pearson’s correlation test. Remaining hits were further analyzed on effects of the response status of donor-patients on the gene expression levels in more details. Linear mixed effects (LME) model with respect to the replicated block design of the experiment was conducted. The continuous gene expression levels were modelled as a combination of fixed and random effects thereby adjusting for possible confounders. Age, gender, depression baseline score, and the response/remission status were treated as fixed effects for the cell lines whereas the experimental units, incubation and time of measurement were included as nested random effects. The random effects were modelled with variable intercept but without variable interaction effects for the experimental factors treatment and time of measurement to avoid over-parameterization. The Model design was confirmed by visual inspection of the trellis plots of the gene expression levels and by Akaike information criterion (AIC) indicating no benefits in case of modeling additionally the slope of the experimental factors. Tests were performed with R v3.5.1 including the libraries coin v1.1-2 and survival 2.39-5 (R Foundation for Statistical Computing, Vienna, Austria). Data are presented as mean ± SEM unless otherwise indicated.

## Results

### Variability in genome-wide gene expression profiling in LCLs of clinical responders and non-responders after incubation with citalopram: exploratory cohort

Whole-transcriptome was studied in LCLs from exploratory SSRI-treated MARS patients who were clinically characterized as responders (≥50% decrease in HAMD score), and non-responders (<50% decrease in HAMD score) after 8-week SSRI-treatment (*n* = 17; 9 RESP, 8 NR). Details on patients’ characteristics are given in Table 1. For biomarker analyses, LCLs from patients were cultivated and gene expression profiles were measured at baseline and after incubation with CTP for 24 and 48 h. Data were analyzed using a hypothesis-free and a pathway-guided algorithm designed to detect cardinal and reactional transcriptomic differences, respectively, between the two response subgroups.

### Hypothesis-free analysis in exploratory MARS cohort

**Fig. 2:**
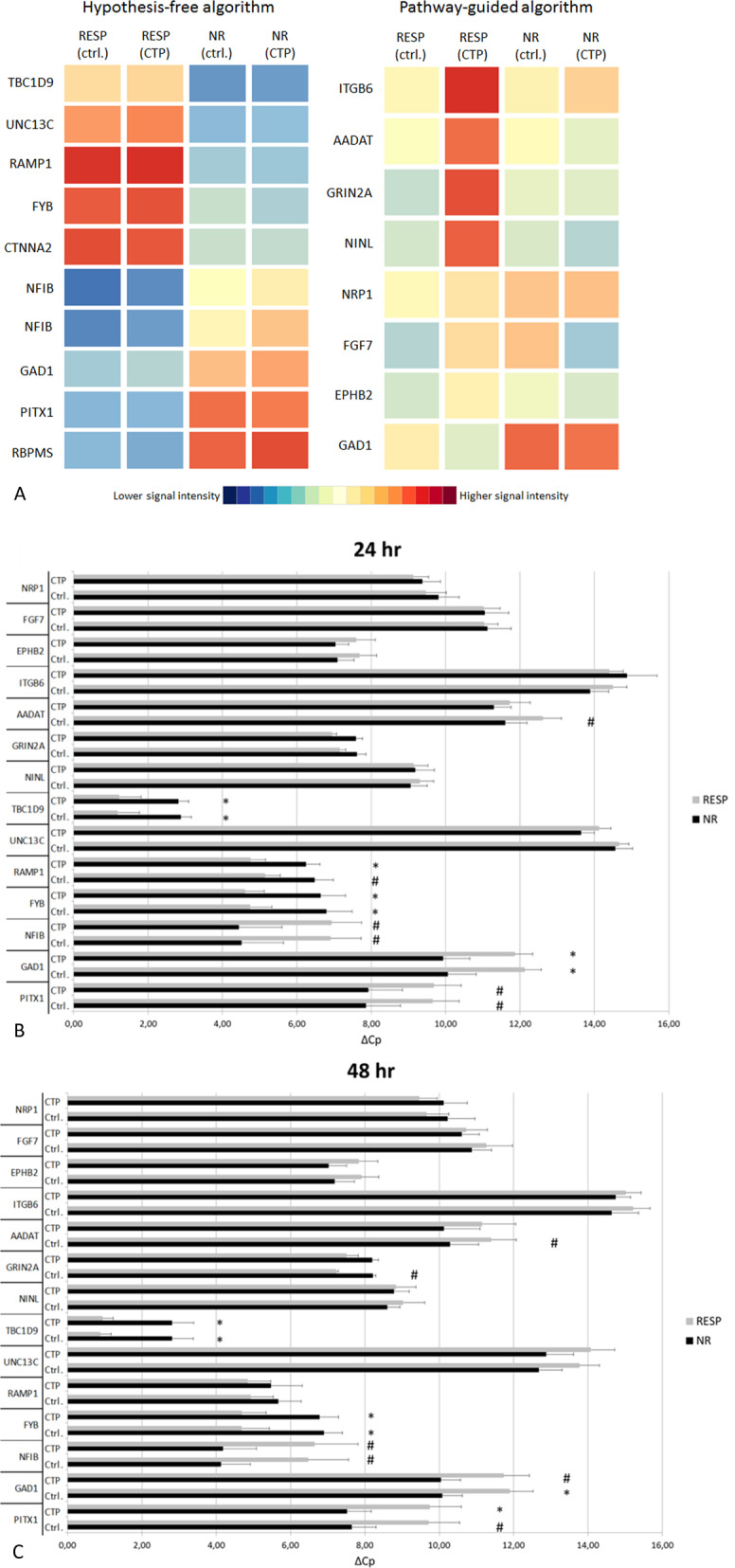
Expression of the candidate genes obtained from the whole-transcriptome profiling in the exploratory MARS cohort (*n* = 17; 9 RESP, 8 NR): shown as a Heat-Map of the microarray data (**a**) and as ΔCp values after 24 (**b**) and 48 (**c**) hours of incubation with CTP (*TBP* -normalized qPCR measurements; mean ± SEM). Expression of *RBPMS* and *CTNNA2* was below the detection limit. *NB* lower ΔCp indicates higher expression; *p ≤ 0.05, ^#^*p* ≤ 0.2 by unpaired *t*-test.

In the hypothesis-free algorithm 55 and 28 genetic features were found significantly differentially expressed between responders vs. non-responders after 24 and 48 h, respectively (FC ≥ 2, *p*-corr. ≤ 0.05; Supplementary Table 4). Twenty-one features were consistently differentially expressed (FC ≥ 2, NR/RESP) over 24 and 48 h. Cardinality was ensured by computing a mean FC value averaged over incubations and time points for each gene. After ranking for calculated mean FC values, top ten features, coding for nine genes (Fig. 2a; Table 2), were considered candidate for qPCR validation.

**Table 2:** Cardinal and reactional candidate genes obtained from the whole-transcriptome profiling using hypothesis free and pathway-guided algorithms.

Hypothesis-free algorithm			
Gene	Averaged FC (NR/ RESP)	*p*-corr. value	Functional relevance
*TBC1D9*	−3.59	0.043	Linked to ADHD^46^.
*UNC13C*	−4.07	0.049	Might be involved in PTSD^75^.
*RAMP1*	−4.63	0.043	Activity modifying protein for CGRP-receptor^76^. CGRP has been linked in depression patients CSF and plasma^77^.
*FYB*	−3.49	0.043	Adapter for FYN, which in turn was associated with long term potentiation^78^.
*CTNNA2*	−3.55	0.043	Found related to bipolar disorder^79^.
*NFIB*	+3.75	0.049	Found involved in depression and antidepressant effects in animal models^41^.
*GAD1*	+3.10	0.020	Linked to depression and antidepressant response^29,30^.
*PITX1*	+4.12	0.049	SNPs were associated with autism^80^
*RBPMS*	+6.47	0.047	Loss of function leads to decreased arborization of axons^81^.

Pathway-guided algorithm			
Gene	Averaged FC (NR/ RESP)	*p*-corr. value	Functional relevance
*NRP1*	+2.75	0.046	Upregulated in postmortem brains from depressed patients^82^.
*FGF7*	−2.10	0.013	Involved in inhibitory synapse formation^83^.
*EPHB2*	+2.36	0.018	linked to depression-like behaviors in animal models^84^.
*ITGB6*	−2.40	4.66E−03	Linked to antidepressant response in MARS cohort^9^.
*AADAT*	+2.60	9.44E−03	Polymorphism was found to modulate SSRI response^85^.
*GRIN2A*	−2.69	3.05E−04	Found to be hypermethylated in the hippocampus of MDD patients^86^.
*NINL*	−2.75	0.031	Quantitative traits-associated susceptibility loci for brain development^87^.
*GAD1*	+3.10	0.043	Expression and variants were linked to depression and antidepressant response^29,30^.

Fold-change differential expression in the response groups and functional relevance of each gene are shown.

### Pathway-guided analysis in exploratory MARS cohort

CTP effects on gene expression were analyzed using the pathway-guided algorithm. After 24 h, CTP altered the expression of 94 and 185 and after 48 h of 1198 and 158 features in NR and RESP, respectively (Supplementary Tables 5, 6). Pathway-enrichment analysis revealed 25 different enriched pathways (*p* ≤ 0.05) in each response group after 24 h incubation, while after 48 h 139 and 14 pathways were enriched in NR and RESP, respectively (Supplementary Table 7). The top 10 significant enriched pathways altered in RESP were involved, among others, in neurotransmitter metabolism, drug addiction, Parkinson’s disease, neuroprotection, and serotonin receptor signaling. On the other hand, the most significant pathways in NR were involved in cellular adhesion and junction, integrin interactions, in addition to immunological pathways like signaling through T-cell receptor, B-cell receptor, IFNγ, CD28 co-stimulation and MHC-II antigen presentation (Table 3).

**Table 3:** Ten most significantly deregulated pathways in responders (*n* = 9) and non-responders (*n* = 8) LCLs from the exploratory SSRI-treated patients after incubation with CTP.

Deregulated pathways in RESP	*p-*corr. value	Deregulated pathways in NR	*p-*corr. value
Hs_Role_of_Osterix_and_miRNAs_in_tooth_development_WP3971_91525	1.17E−03	Hs_Focal_Adhesion_WP306_94849	3.60E−10
Hs_Elastic_fibre_formation_WP2666_76849	0.005	Hs_Integrin_cell_surface_interactions_WP1833_77019	6.68E−10
Hs_Hypothetical_Network_for_Drug_Addiction_WP666_68893	0.005	Hs_Vitamin_D_Receptor_Pathway_WP2877_94793	8.02E−10
Hs_Melatonin_metabolism_and_effects_WP3298_91618	0.005	Hs_MHC_class_II_antigen_presentation_WP2679_76872	1.38E−09
Hs_Neurotransmitter_Receptor_Binding_And_Downstream_Transmission_In_The_Postsynaptic_Cell_WP2754_77001	0.007	Hs_TCR_signaling_WP1927_76950	1.41E−09
Hs_Parkinsons_Disease_Pathway_WP2371_87374	0.007	Hs_TYROBP_Causal_Network_WP3945_90843	3.45E−09
Hs_NO-cGMP-PKG_mediated_Neuroprotection_WP4008_92677	0.009	Hs_Interferon_gamma_signaling_WP1836_77096	3.96E−09
Hs_Tryptophan_metabolism_WP465_94086	0.009	Hs_Cell_junction_organization_WP1793_77057	4.23E−09
Hs_Serotonin_Receptor_2_and_STAT3_Signaling_WP733_74441	0.013	Hs_Costimulation_by_the_CD28_family_WP1799_77064	4.84E−09
Hs_Lung_fibrosis_WP3624_92327	0.013	Hs_B_Cell_Receptor_Signaling_Pathway_WP23_92558	5.00E−09

For candidate gene selection, 40 genetic features enriched in pathways deregulated in responders (*p* ≤ 0.05) were considered. Features were then filtered on differential expression in response groups (FC ≥ 2 NR/RESP). Out of the resulting 14 features, 8 genes with functional relevance were considered for further qPCR validation (Fig. 2a; Table 2). Interestingly one gene, *GAD1*, emerged as a hit in both the hypothesis-free and the pathway-guided algorithm.

### qPCR validation of the candidate genes in MARS cohort (*n* = 69)

After identifying candidate genes, microarray results were validated via qPCR upon incubation with CTP and ctrl. for 24 and 48 h. Firstly, in the exploratory MARS cohort 4 out of the 16 candidate genes (from 18 features), showed significantly different expressions (*p* ≤ 0.05) between the response groups at least under one of the incubation conditions: glutamate decarboxylase 1 (*GAD1*), FYN binding protein (*FYB*), receptor activity modifying protein 1 (*RAMP1*), TBC1 domain family member 9 (*TBC1D9*), while other four genes showed a trend (*p* ≤ 0.2): paired like homeodomain 1 (*PITX1*), nuclear factor I B (*NFIB*), glutamate ionotropic receptor NMDA type subunit 2A (*GRIN2A*), aminoadipate aminotransferase (*AADAT*) (Fig. 2b, c, Supplementary Table 8). Expression of RNA-binding protein with multiple splicing (*RBPM*S) and catenin alpha-2 (*CTNNA2*) was below the detection limit (Cp values > 40).

Expression of the resulting 8 candidate genes was further investigated in LCLs from MARS validation cohort of depression patients characterized for response upon treatment with a serotonin-transporter-inhibiting antidepressant of the classes SSRIs, SNRIs and TCAs (*n* = 69; 33 RESP, 36 NR). Bivariate statistics (Welch’s *t*-test) showed an association of *TBC1D9* with remission status under all incubation conditions (*p* = 0.014–0.021) while *GAD1* showed marginal to significant association with response status (*p* = 0.038–0.069, Supplementary Table 9). Pearson’s analyses showed significant correlations between *NFIB* expression (∆Cp) and clinical improvement (*p* = 0.015–0.025, *r* = −0.123 to −0.295, Supplementary Table 10). In addition to the bivariate statistics, clinical response and remission were tested for effects on gene expression in a linear mixed-effects (LME) model taking baseline depression severity (baseline HAMD score), gender, age, incubation and incubation period as covariates. The multivariate analysis showed an association of *GAD1* expression with response status (*p* = 0.045) but only a tendency toward association with remission status (*p* = 0.088). Expression of *TBC1D9* and *NFIB* could not reach significance for association with response (*p* = 0.63 and 0.53, respectively) nor with remission (*p* = 0.29 and 0.181, respectively).

### Expression of the candidate genes in response edge groups of the STAR*D study

After identifying candidate biomarkers for antidepressant response in MARS cohort, we tested the 8 candidate genes from the whole-transcriptome analysis for validity in differentiating patients from the two clinical extremes: first-line responders and treatment-resistant patients. Expression of *GAD1, FYB, RAMP1, TBC1D9, PITX1, NFIB, GRIN2A*, and *AADAT* was validated after incubation with citalopram (3 µM, for 24 and 48 h) with qPCR in LCLs from 24 first-line-treatment responders and 20 treatment-resistant patients from the STAR*D cohort. While bivariate tests could not detect associations with clinical outcomes (Supplementary Table 9), multivariate analysis showed a remarkable tendency toward association with the expression of *NFIB* (*p* = 0.068). *TBC1D9* and *GAD1* expression showed no association with treatment resistance (*p* = 0.27 and 0.23, respectively).

## Discussion

Early identification of patients expected not to respond to specific antidepressant classes would help in early decision on treatment with other antidepressants or therapeutic interventions. In search for tentative, early biomarkers for antidepressant outcome we studied transcriptional differences in LCLs from depression patients documented for clinical response status to serotonin-transporter-inhibiting antidepressants upon in-vitro incubation with citalopram (CTP). Studies on drug utilization in Europe showed serotonin-transporter-inhibiting antidepressants (SSRIs, SNRIs, and TCAs) to be the most frequently prescribed antidepressant classes^16–18^ with citalopram leading antidepressants prescribed in Germany^19,20^. LCLs use for investigating functional biomarkers for antidepressant response prediction has been established^9–11^. However, in contrast to earlier works, we focused on studying short-term transcriptional changes occurring after 24 and 48 h of in-vitro incubation with CTP. Differential expression regulation persistent through both-time points should indicate higher robustness of identified candidate genes. CTP concentration used (3 µM) was 10-fold higher than therapeutic plasma concentration^21^ in line with earlier studies on antidepressants in LCLs^22^. However, being a racemic mixture of the active S- and inactive R-enantiomers, the concentration is comparable to brain–blood ratios reported in rodents^23^ and humans^24^. Moreover, in-vitro data suggest that CTP concentration of 12.5 µM up to 96 h are not cytotoxic for LCLs (Supplementary Methods 2). Whole-transcriptome data were analyzed using a hypothesis-free and a pathway-guided algorithm designed to detect cardinal and reactional transcriptomic differences, respectively, between the responding and non-responding patients-derived LCLs.

### Identification of transcriptional antidepressant response biomarkers

Cardinal and reactional transcriptomic differences between the responders and non-responders of MARS exploratory cohort were determined and 16 candidate genes were selected. Interestingly, top features from the hypothesis-free algorithm have been previously reported to be involved in neuropsychiatric disorders (see Table 2). The emergence of *GAD1* as a hit in both algorithms implies a major role in depression pathophysiology as well as in the serotonergically mediated antidepressant action. This suggestion was further supported by the qPCR validation results. GAD1 expression was associated with response status and, to less extent, with remission in MARS exploratory and validation cohorts. *GAD1* encodes glutamate decarboxylase 1, an enzyme responsible for the last and rate-limiting step in the synthesis of the inhibitory neurotransmitter GABA from the excitatory glutamate^25^. This suggests that GAD1 may act as a link between the two opposing circuits in the brain. GABA and glutamate control the vast majority of inhibitory and excitatory signaling in the brain and, hence, influence emotional stability and control the pathophysiology of mood disorders^26^. The GABAergic signaling was also found to tightly control the hypothalamic-pituitary-adrenal (HPA) axis which is known to mediate the body’s neuroendocrine response to stress, and there is growing evidence that GABAergic imbalance exacerbates stress impact on depression^27^. Indeed, interaction between rs769407 of *GAD1* and rs173365 of the corticotrophin-releasing-hormone-receptor 1 *CRHR1* gene was described in a subgroup of depression patients with sleep disturbance symptoms^28^. The antidepressant response has also been found to correlate with *GAD1* genetic variants. A SNP in *GAD1*, rs11542313, was found in interaction with two other SNPs in GABAA receptor subunits delta and epsilon genes, *GABRD* and *GABRE*, modulating antidepressant therapeutic response^29^. Expression studies found reduced *GAD1* expression in brain samples from depression patients^30,31^. However, *GAD1* expression in blood showed an opposite regulation to that in the brain. Lin et al. found increased GAD1 in drug-naïve patients than in medicated patients and healthy individuals^32^. Suitably, our results of higher expression of *GAD1* in non-responders LCLs are in a better accordance with results from peripheral lymphocytes than with those from brain samples.

*NFIB* expression showed significant correlations with clinical improvement as tested with Pearson’s correlation in MARS cohort. LME analysis did not, however, detect associations with response or remission status. *NFIB* belongs to the nuclear factor one (NFI) family, a transcription factors family essential for normal development of several organs including central nervous structures like the spinal cord^33^, cerebellum^34^, and the hippocampus^35^. Specifically, NFIB coordinates gliogenesis in embryos^33^ and stimulate the differentiation of astrocytes^36,37^ and oligodendrocytes^38^. Glial cells implication in MDD raised from observations of their decreased numbers in post-mortem samples from regions of the brain including hippocampus^39^ and prefrontal cortex (PFC)^40^ in depressed patients. *NFIB* expression has been related to the HPA axis function as animal studies found that chronic mild stress increased *NFIB* expression in the frontal cortex in rats and in the PFC and amygdala in mice while pharmacological treatment normalized *NFIB* levels^41–43^. Moreover, NFIB expression was found to be responsive to changes in plasma cortisol concentration^44^.

Expression of *TBC1D9* was stably associated with remission status in MARS cohort und both incubation status and time points. Similar to *NFIB*, the association did not survive correction for covariates in LME model. TBC1D9 belongs to the Tre‐2/Bub2/Cdc16 (TBC) domain-containing proteins family. Members of this family possess a GTPase-activating activity with affinity to the Rab family, the largest intracellular membrane trafficking proteins family in eukaryotes^45^. Little is known on the function of TBC1D9. Relation to neuropsychiatric disorders emerged as a de-novo missense mutation (His1179Tyr) in *TBC1D9* was observed in individuals with ADHD cases lacking family history^46^. Further members of TBC family were repeatedly found to be involved in intraneuronal vesicle trafficking and, hence, in neuropsychiatric disorders. TBC1D12, for example, was found to modulate neuron morphology by stimulating neurite formation^47^. Links to depression have also been previously reported. Expression of *TBC1D10C* and *TBC1D5* in blood has been found elevated in mice^48^ and patients^7^, respectively.

In order to investigate potential resistance-related molecular profiles, an independent cohort of patients-derived LCLs was analyzed which reflects the extremes of treatment resistance and primary response to the first try with CTP as an antidepressant drug. Analyzing candidate genes in this cohort showed solely a tendency toward an association of *NFIB* expression with the treatment resistance. Associations with *GAD1* and *TBC1D9* were not replicated in the STAR*D cohort. These findings come in line with the growing evidence of lacking or partial overlap between biomarkers tested for different clinical outcome phenotypes. Our previous investigations on neuroplasticity biomarkers for antidepressant response identified cell proliferation to be predictive for treatment-resistance status, but not for response status. Moreover, whole-transcriptome biomarkers for treatment resistance were only partially reproducible for response. While *WNT2B, ABCB1*, and *FZD7* expression correlated with treatment-resistance status, expression of *WNT2B, SULT4A* correlated with response status, suggesting *WNT2B* as a common predictor^10,11^. The few available results on genetic biomarkers draw up a similar course. The European Group for the Study of Resistant Depression (GSRD) correlated variants of rs10501087 and rs6265 in *BDNF* to non-response, while *5HTR2A* rs7997012 and *CREB1* rs7569963 were found to correlate with treatment resistance (reviewed by Schosser et al.^49^). Likewise, in a prospective study with 220 depression patients characterized for response, remission, non-response, non-remission, and treatment resistance, Fabbri et al. identified different genetic variants for each phenotype of treatment outcome. Only MAPK1 rs6928 G/GG-alleles additively associated with better treatment outcomes, response and remission, respectively^50^. Although a later meta-analysis of three independent samples (n_total_ = 3225) found no single genetic predictor, two gene-sets (GO:0000183 chromatin silencing and GO:0043949 regulation of cAMP-mediated signaling) were enriched in treatment resistance versus the compiled group of responders and non-responders^2^. Our results, taken together with earlier finding, suggest the existence of distinct neurobiological etiologies of different treatment outcomes.

Noteworthy is the absence of findings from our earlier research^9–11^ in the hits from the current transcriptome-wide analysis. This absence might be attributed to several aspects that mark up the present study. First, earlier findings were largely driven by the proliferation rate of LCLs as a surrogate ex-vivo biomarker for neuroplasticity, while the current study stratified donor patients on diagnostic (only unipolar depression) and therapeutic (SERT-inhibiting ADs) profiles to obtain an as homogenous cohort as feasible. Secondly, the incubation time in earlier studies was 3 weeks, resembling the usual time needed for reliable evaluation of treatment efficacy^51^, whereas in this study cell lines were incubated for 24 and 48 h in an approach closer to real-life biomarker applicability.

### Pathway analysis of CTP-deregulated features in RESP and NR

Our results from the exploratory cohort (MARS) indicate a profoundly response-status-dependent transcriptional reaction to short-time incubation with CTP in LCLs. While the reaction in responders’ cells highlighted involvement of neurotransmitters metabolism, serotonin receptor and other neurological and neuropathological pathways, non-responders cells showed less neural-specific reaction with most significant pathways being involved in cell adhesion and immune response. Unlike the hypothesis-free analysis in which straight comparisons between gene expression levels in the two response groups (RESP/NR), the pathway analysis focused on genes that were deregulated by CTP incubation in each response group (CTP/ctrl.). Pathway analysis have been suggested to increase power in detecting associations with antidepressant response in comparison to approaches studying single genetic signatures^52,53^. However, lack of solid findings in antidepressant pharmacogenetic studies might have led to fewer reports on pathways associated with antidepressant response^54^. Additionally, very few studies investigated the pathway regulation elicited by antidepressants in association with the clinical outcome. In an earlier proteomic analysis of mononuclear cells in depression patients before and after 6-week antidepressant treatment, de Souza et al. suggested that antidepressant medication affects similar biological pathways in responders and non-responders but in different direction^6^. This seems to come in conflict with our results of RESP and NR deregulating different pathways in reaction to CTP. Several technical differences can explain the discrepancy in results. While our study focused on identifying applicable, short-term biomarkers in homogenous cell lines derived from one leucocyte subtype, the B lymphocytes, de Souza et al. investigated mononuclear cell populations after a long-term 6-week antidepressant therapy. Additionally, our study did not consider post-transcriptional events, including the proteome, which was in focus of de Souza’s study.

The findings of neurotransmitters metabolism pathways (dopamine, serotonin, and other biogenic amines) recall the conventional hypothesis on involvement of neurotransmitter imbalance as an underlying biological mechanism of depression^55^. The hypothesis emerged in parallel to the successful use of monoamine oxidase inhibitors (MAOi) in managing depression before deprioritizing them in later guidelines due to drug–food and drug–drug interactions^56^. In line with our results, a previous pharmacometabolomic study reported involvement of neurotransmitter metabolic pathways in response to the SSRI sertraline. Peripheral baseline levels of dihydroxyphenylacetic acid (DOPAC), a metabolite of dopamine, and serotonin, seen as a metabolite of tryptophan, were found among other metabolites with a binary response-discriminant ability^57^. Further studies identified alterations in expression of serotonin biosynthesis pathway genes to correlate with SSRI response^58^ and decreased metabolism of tryptophan to be associated with response to ketamine^59^.

There is growing evidence that the immune system plays a major role in depression pathophysiology and therapy response which resulted in the emergence of the immunological hypothesis of depression^60^. Genetic variants and expression of inflammatory blood markers including chemokines, cytokines, and acute-phase proteins were associated with depression and/or poor therapy response^61–63^. In this context, our results come in analogy with previous findings of B cell receptor signaling pathway being associated with clinical outcome in two independent cohorts of depression patients^64^. Further studies reported increased soluble IL-2 receptor, a T cells activity marker, in peripheral blood in depression patients^65^. Treatment with interferons, on the other hand, was repeatedly found to induce depressive episodes^66–68^.

Cellular adhesion molecules (CAMs) are cell surface proteins involved in cell–cell or cell–extracellular matrix binding, a process important for immune response, inflammation, and neurogenesis^69^. Polymorphisms and expression of several CAMs were linked to autistic spectrum disorders^70^, schizophrenia^71^ and depression. In a large-scale GWAS (*n* = 3394) investigating molecular mechanisms involved in depression etiology cell adhesion molecules and focal adhesion pathways were found to be among the top 5 enriched pathways. Further pathways were found related to neurotransmitters and the immune system^72^. Lately, two CAMs, *CHL1* and *ITGB3*, were repeatedly reported to be candidate predictors of antidepressant response. Associations between SNPs rs4003413 (*CHL1*) and rs3809865 (*ITGB3*) with response could be significantly replicated in two independent depression samples^73^ while expression data found associations with early remission^9^.

Results from animal studies on pathway regulation in response models are spars. In a study in rats, animals that responded to escitalopram treatment after chronic mild stress showed differential expression to non-responding littermates in genes related to apoptosis, hippocampal neurotransmission and TNF signaling, coming in good accordance with our enriched pathways^74^.

Thus, our findings show that deregulated pathways underline a molecular profile of antidepressant drug effects that differs between responders and non-responders (Top 10 significantly enriched pathways in RESP involved in neurotransmitter metabolism, drug addiction, Parkinson’s disease, neuroprotection, and serotonin receptor signaling, while in NR most significant pathways involved in cellular adhesion, integrin interactions, in addition to immunological pathways). Although altered pathways in both response groups are involved in depression biology and/or antidepressant response mechanism, the complex, less specific reaction seen in NR could either imply a more complicated underlying molecular pathophysiology in these patients or an indefinite reaction to antidepressant therapy in which some pathways oppose the sought healing effects provoked by the others.

It should be noticed, however, that unlike the expression of the candidate genes which was validated in a larger sample size using a distinct technical methodology and multivariate statistical analyses corrected for, among others, age and gender, data from the pathway analysis were based on a limited sample size which was unbalanced for gender.

### Limitations

Several limitations have to be considered while interpreting our results. First, the gene-expression profiling was conducted in a homogenous in-vitro model based on patients-derived LCLs. Nevertheless, results in patients might deviate from that observed from in vitro cell models. A further limitation that should be seriously considered is the heterogeneity of the MARS cohort which results from its observational character. Although we tried to address this problem with a tight stratification before gene expression profiling, we cannot rule out that effects in gene expression might be overlooked due to this fact.

## Conclusion

To our best knowledge, this is the first study to investigate treatment-outcome-predicting transcriptional biomarkers in depression patients recruited in two independent studies. Our results suggest that biological pathways reacting to citalopram are different in responding and non-responding patients LCLs. In our biomarker analysis, expression of *GAD1*, *NFIB*, and *TBC1D9* showed associations with response status, remission status, and improvement in depression scale, respectively, but not with treatment resistance. This supports the notion of the existence of distinct neurobiological etiologies of different treatment outcomes and stresses the emerging need to decipher the molecular mechanisms and biomarkers in the different clinical outcomes phenotypes severally. Being functionally involved in the glutamatergic/GABAergic systems and in neurogenesis, GAD1, NFIB, and TBC1D9 are promising candidates for further pharmacogenetic variability studies in larger patients cohorts.

## Supplementary information

Supplementary Methods 1

Supplementary Methods 2

Supplementary Tables
