# Supplementary material for: Citalopram-induced pathways regulation and tentative treatment-outcome-predicting biomarkers in lymphoblastoid cell lines from depression patients
Source: Transl Psychiatry. 2020 Jul 1;10:210. doi: 10.1038/s41398-020-00900-8 (PMC7329820; doi:10.1038/s41398-020-00900-8)
Supplement: Supplementary file 1 — Supplementary Methods 1 [file 41398_2020_900_MOESM1_ESM.docx]

# Supplementary Methods 1

## Generation of lymphoblastoid cell lines

LCLs were derived from blood samples through Epstein–Barr virus transformation 17,18. Peripheral blood mononuclear cells (PBMCs) were firstly isolated by Ficoll gradient, resuspended in Epstein–Barr virus supernatant from B95-8 cell culture, and 100 μl were seeded into 8 wells 48-well cell culture plate. Each well received 200 μl RPMI-1640 medium (containing 20% fetal bovine serum FBS) and the plate was incubated at 37°C in a humidified CO2 incubator (with 5% CO2) for 5 days. Subsequently, 300 µl culture medium + cyclosporine (1 μg/ml) were added to each well. After 25 days, wells were pooled and the cell surface markers was identified using the CD3, CD19 and CD45 antibodies (BD Tritest Kit, Becton Dickinson, Heidelberg, Germany) in flow cytometry. The cells were cryo-stored in 90% FCS and 10% DMSO in liquid nitrogen (-186°C) no longer than 5 years.

## Cell culture and nucleic acid isolation

LCLs (from both cohorts) were cultured in RPMI-1640 medium supplied with L-glutamine (2 mM), penicillin G sodium/streptomycin sulfate mixture (60.27 and 100 µg/ml, respectively; Biowest, France) and 15% heat-inactivated FBS (Biochrom, Germany). Cells were fed three times a week and incubated in a humidified incubator at 37 °C and 5% CO_2_. A 10-mM stock solution of citalopram-HBr (Sigma-Aldrich, Germany) was prepared in 96% ethanol (Merck-Millipore, Germany). Cell lines were cultivated for no longer than 2 weeks before incubation with citalopram (CTP). Experiments were conducted by incubating 1*10^7^ cells cultivated in 20 ml medium with CTP in a final concentration of 3 µM. CTP concentration used was in line with earlier studies on short-term incubation of LCLs with SSRIs and below cytotoxic concentration measured earlier by XTT assay (see supplementary Methods 2). Controls received equal volume of ethanol achieving a final concentration of 0.03% v/v. LCLs from remitters, responding, non-responding and treatment-resistant patients were incubated in parallel. Contamination with mycoplasma was periodically tested using MycoAlert™ Plus ELISA kit (Biozyme, Oldendorf, Germany). After 24 and 48 hours, samples were collected, centrifuged and cell pellets were washed with PBS and stored at -80°C. RNA was isolated using Nucleospin® RNA purification kit (Macherey-Nagel, Düren, Germany). RNA concentration and A260:280 ratio was measured using NanoDrop™ 1000 (Thermo Scientific, Darmstadt, Germany). RNA integrity number (RIN) was spot-checked as a further quality control measure using 2100 Bioanalyzer (Agilent Technologies, Waldbronn, Germany). In this study RNA used had A260:280 ratio >1.9 and RIN >9.6.

## Genome-wide expression analysis

Whole-genome expression analyses were done using Agilent SurePrint Human GE V3 8x60K microarrays containing probes for >56 000 genetic entities coding for >40000 unique transcripts. RNA was labeled using Low Input Quick-Amp Labeling Kit (Agilent Technologies) according to the kit manual. Briefly, 100 ng RNA were reversely transcribed to cDNA, from which Cy3-labeled cRNA was transcribed, purified and hybridized to the expression microarray. Hybridization, scanning, and data extraction from scanned microarrays were conducted according to the delivered protocol.

## Quantitative PCR validation of candidate genes in MARS and STAR*D cohorts

Expression of candidate genes identified with whole-genome expression analysis was validated using qPCR. Firstly, cDNA was reversely transcribed from 1 µg RNA using Transcriptor First Strand cDNA Synthesis Kit (Roche, Mannheim, Germany) according to the kit manual. Gene expression was quantified using QuantiTect® SYBR Green PCR kit (QIAGEN, Hilden, Germany) in LightCycler 480 II (Roche) in technical duplicates. All Primers were purchased from the QuantiTect® Primer Assay pipeline except the primer pair for AADAT which was purchased from RT² qPCR Primer Assay pipeline (pipelines by QIAGEN). Gene expression was calculated using ΔCp normalized to *TBP*.
