# Supplementary material for: Citalopram-induced pathways regulation and tentative treatment-outcome-predicting biomarkers in lymphoblastoid cell lines from depression patients
Source: Transl Psychiatry. 2020 Jul 1;10:210. doi: 10.1038/s41398-020-00900-8 (PMC7329820; doi:10.1038/s41398-020-00900-8)
Supplement: Supplementary file 2 — Supplementary Methods 2 [file 41398_2020_900_MOESM2_ESM.docx]

## Supplementary methods 2

**Determination of citalopram cytotoxic effects using XTT assay**

Cytotoxic effects of different concentrations of CTP were identified upon incubation with LCLs for 24, 48, 72 and 96 hours. For this test XTT cell viability assay kit (ATCC®) was used. The principle of the assay depends on measuring the cell viability by measuring the mitochondrial oxireductases activity. XTT is reduced at the cellular membrane from a pale yellow to red-orange, water-soluble compound. The generation of the color is proportional to the metabolic activity^1^. Cells were cultivated in four 96-well plates, one for each time point. =2 * 10^4^ cells were added to each well. Afterwards, 10 μl of the corresponding working solution (prepared in culture medium) were added to the wells in triplicates to achieve final concentrations between 12.5 and 300 µM. Medium and cell suspension controls (0 and 100% cell viability) were pipetted in 6 replicates each and received 10 μl medium. After 24, 48, 72 and 96 hours of incubation 50 μl freshly activated XTT reagent were added to each well, including the controls, and incubated for 4 hours at 37°C. Safire^2^ plate reader (Tecan) was used for measuring the absorbance at 475 nm. Half maximal inhibitory concentration (IC_50_) of citalopram was calculated for each time point using Quest Graph™ with help of the following equation^2^:

$$Y = Min + \frac{Max - Min}{1 +(\frac{X}{{IC}_{50}})^{Hill coefficient}}$$

Where:

*X* is the concentration of CTP (µM)

*Y* is cell variability (% of control)

*Max* maximum tested CTP concentration (µM)

*Min* minimum tested CTP concentration (µM)

*Hill coefficient* is a measure of ultra-sensitivity (i.e. how steep is the response curve)

Cell viability shown in percentage of control (SD) of LCLs upon incubation with citalopram (12.5-300 µM) for 24, 48, 72 and 96 hours.

| CTP (µM) | 24 hr | 48 hr | 72 hr | 96 hr |
| --- | --- | --- | --- | --- |
| 0 | 100.0 (9.21) | 100.0 (4.93) | 100.0 (10.21) | 100.0 (13.53) |
| 12.5 | 110.6 (10.73) | 113.4 (4.05) | 108.4 (7.02) | 99.4 (10.33) |
| 25 | 89.2 (7.90) | 98.1 (7.22) | 77.3* (5.82) | 84.7 (8.80) |
| 50 | 59.2* (12.15) | 63.4* (3.66) | 50.7* (4.26) | 47.2* (3.17) |
| 100 | 25.5* (3.64) | 16.6* (3.48) | 8.1* (0.55) | 2.7* (0.81) |
| 150 | 9.5* (2.03) | 5.0* (0.74) | 5.1* (0.30) | 1.0* (0.88) |
| 200 | 0.0* (2.22) | 2.7* (1.44) | 4.7* (0.11) | 0.4* (0.23) |
| 300 | 0.0* (4.82) | 3.7* (0.57) | 4.3* (0.16) | 0.0* (0.75) |
| IC_50_ Calculation | | | | |
| IC_50_ (µM) | 59.5 | 55.8 | 44.7 | 47.6 |
| Hill coefficient | 2.166 | 3.265 | 2.519 | 3.194 |

* significantly different from control (*p*≤0.05)

Cell viability shown in percentage of control (SD) of LCLs upon incubation with citalopram (12.5-300 µM) for 24 (blue diamonds), 48 (red squares), 72 (green triangles) and 96 (purple crosses) hours.

**References:**

1. D.A. Scudiero et al. (1988). "Evaluation of a soluble tetrazolium/formazan assay for cell growth and drug sensitivity in culture using human and other tumor cell lines." Cancer Res.(48): 4827-4833.
2. AAT Bioquest, Inc. (2015, December 18). Quest Graph™ IC50 Calculator.". Retrieved from https://www.aatbio.com/tools/ic50-calculator.
